# NMR Methods for Determining Lipid Turnover via Stable Isotope Resolved Metabolomics

**DOI:** 10.3390/metabo11040202

**Published:** 2021-03-29

**Authors:** Penghui Lin, Li Dai, Daniel R. Crooks, Leonard M. Neckers, Richard M. Higashi, Teresa W-M. Fan, Andrew N. Lane

**Affiliations:** 1 Center for Environmental and Systems Biochemistry, University of Kentucky, 789 S. Limestone St, Lexington, KY 40536, USA; penghui.lin@uky.edu (P.L.); Rick.higashi@uky.edu (R.M.H.); twmfan@gmail.com (T.W-M.F.); 2 Urologic Oncology Branch, Center for Cancer Research, National Cancer Institute, National Institutes of Health, Bethesda, MD 20892, USA; joycedaili@gmail.com (L.D.); crooksda@mail.nih.gov (D.R.C.); neckersl@mail.nih.gov (L.M.N.); 3 Department Toxicology & Cancer Biology, University of Kentucky, 789 S. Limestone St, Lexington, KY 40536, USA

**Keywords:** stable isotope tracers, Nuclear Magnetic Resonance, lipid ^13^C incorporation, isotopomer distributions

## Abstract

Lipids comprise diverse classes of compounds that are important for the structure and properties of membranes, as high-energy fuel sources and as signaling molecules. Therefore, the turnover rates of these varied classes of lipids are fundamental to cellular function. However, their enormous chemical diversity and dynamic range in cells makes detailed analysis very complex. Furthermore, although stable isotope tracers enable the determination of synthesis and degradation of complex lipids, the numbers of distinguishable molecules increase enormously, which exacerbates the problem. Although LC-MS-MS (Liquid Chromatography-Tandem Mass Spectrometry) is the standard for lipidomics, NMR can add value in global lipid analysis and isotopomer distributions of intact lipids. Here, we describe new developments in NMR analysis for assessing global lipid content and isotopic enrichment of mixtures of complex lipids for two cell lines (PC3 and UMUC3) using both ^13^C_6_ glucose and ^13^C_5_ glutamine tracers.

## 1. Introduction

Lipids comprise an extremely diverse group of compounds that have a wide range of biological functions from structure (e.g., membranes) to signaling molecules. Even discounting all the many ways in which they form complexes with other molecules, lipids come in an enormous diversity of forms [1,2]. These range from fatty acids as short as acetate (*n* = 2) to palmitic acid, the end product of fatty acid synthase activity (*n* = 16), to longer chain fatty acids such as arachidonic acids (*n* = 24), which are incorporated into complex lipids via esterification to a variety of headgroups (Figure S1, Scheme S1). The natural fatty acids may be saturated or have different numbers of double bonds in different positions, and may have either odd or even chain lengths, and in some cases branched chains. A completely separate and diverse branch of lipids also based on acetate is the steroids, which include cholesterol, and their metabolic products, the bile acids. Complex lipids comprise compounds that contain 1–3 fatty acyl chains linked to a short backbone group such as glycerol, which is further esterified to a wide range of head groups. Even for glycerophospholipids with 6 different headgroups and 20 fatty acids, there are thousands of possible lipids (many of which do not occur naturally) [Figure S1]. A significant number of the lipids are also isomers, further complicating analysis, and which is only partly resolved by tandem MS at a very high resolution [3]. It has been estimated that there are more than 1000 natural lipid species present in a eukaryotic cell [4], and all organisms either synthesize them de novo or acquire them from the diet [5]. In mammals, there are a few essential fatty acids such as linoleic and α-linolenic acid, but there are also many lipids present that are of exogenous origin (e.g., cholesterol, triacylglycerols, and fatty acids), and all may be represented in the analytical platforms [6,7,8]. Furthermore, the range of concentrations of various lipids found in tissues is very large. Much of lipidomics has been concerned with identifying which lipids are present in specific biological systems. Although most of this work has been done by mass spectrometry, there are increasingly extensive databases being made available for heteronuclear NMR [9,10], which complements the LC MS/MS methods commonly used.

Metabolomics is a powerful tool for directly accessing molecular level change in cells. With the help of stable isotope labeling, it is possible to follow the fate of individual atoms from precursors into downstream metabolites. However, assessing stable isotope incorporation into complex lipids, especially at low enrichment, has long been a challenge due to the complexity of lipid structures and the vast number of lipid species [11,12]. Nevertheless, modern mass spectrometers are capable of resolving different isotopologues in stable isotope experiments designed to assess lipid turnover in a wide variety of biological systems including tracer studies in vivo [13,14,15,16,17,18,19]. Furthermore, lipid synthesis involves multiple metabolic pathways: both fatty acids and cholesterol derived from AcCoA (Acetyl Coenzyme A) units but in separately regulated pathways, and the glycerol and headgroup subunits of the glycerolipids, involve other pathways such as glycolysis (for glycerol) and amino acid synthesis or uptake (e.g., serine) (cf. Figure S1). Assessing the relative importance of these different pathways and nutrient sources requires stable isotope tracing [20].

Cancers exhibit enhanced nutrient utilization and metabolic reprogramming [21,22,23,24,25,26,27]. As the metabolism is a complex network of coupled chemical reactions that transfer substructures of molecules, the metabolite substructures must be tracked. Stable isotope resolved metabolomics (SIRM) distinguishes pathways, functions, and even chemically identical metabolites by tracking the provenance of substructures via NMR [28]. Beyond pathways, the metabolic networks can be tracked through multiplexed SIRM (mSIRM) experiments [29] which requires higher instrument performance to accommodate its needs.

NMR-based SIRM measurements provide information about positional labeling due to metabolic activity, and thus about pathway utilization by cells under different conditions [23]. This is accomplished by administering metabolic tracers with stable isotope labels to cell or tissue cultures or whole organisms, including rodents and human subjects. We use both multinuclear NMR and UHR-MS (UltraHigh Resolution Mass Spectrometry) to analyze multi-elemental isotopologues [29,30]. Although stable isotope tracing has been widely used for polar metabolites, it has not been extensively applied to lipidomics, which is almost exclusively studied by mass spectrometry [13,20,31,32,33]. Furthermore, with some exceptions, much of the MS-based lipid tracing has used deuterium, such as the incorporation from heavy water in physiological experiments [19,33]. Although NMR is generally much less sensitive than MS, and the resolution of lipid species is low in NMR, there are global features of lipid mixtures than are more readily accessible by NMR than by MS, especially in the context of ^13^C metabolic tracing [20,34,35]. There has been increasing interest in using NMR with stable isotope tracing for lipid analysis, in a diverse range of settings [36,37,38,39,40]. The use of a ^2^H or ^13^C stable isotope tracer or a combination of both for NMR analysis to study *de novo* lipogenesis has been reported [40,41]. However, direct ^2^H or ^13^C detection have low sensitivity, so it is very time-consuming to acquire high-quality spectra compared with ^1^H detection. Although direct ^13^C detection observes non-protiated positions (e.g., carbonyl and quaternary carbons), and generally there is higher resolution, proton detection can be up to 32 times as sensitive as direct ^13^C detection, where a gated decoupling experiment is used for quantitative analysis, thus requiring either more than an order of magnitude less material or up to three orders of magnitude less time for the same signal-to-noise ratio. Similarly, ^2^H has poorer resolution and is up to 100 times less sensitive than ^1^H, though, because of the short T_1_ value, it may be of comparable sensitivity to ^13^C [41].

In particular we compare results using TOCSY (Total Correlation Spectroscopy) and 1D ^1^H{^13^C}-HSQC (Heteronuclear Single Quantum Coherence spectroscopy) for determining ^13^C incorporation into different metabolic subunits of complex lipids (glycerol versus fatty acyl chains in glycerolipids, and methyl groups of cholesterol) in an unfractionated mixture. This study utilizes the methyl resonances of phosphatidyl choline (PC) as an internal reference, which has a known natural abundance level of ^13^C, to normalize other lipid subgroup signals in both 1D proton and 1D HSQC spectra to determine the fractional enrichment. The TOCSY spectra are used for two purposes: (i) signal assignment and (ii) independently determining ^13^C enrichment, at least for the glycerol subunits.

TRAP-1 (TNF (Tumor Necrosis Factor) Receptor Associated Protein-1) is a mitochondrial chaperone protein of the heat shock family. It has been reported that TRAP-1^−/−^ mice showed a reduced incidence of age-associated pathologies, including obesity, inflammatory tissue degeneration, dysplasia, and spontaneous tumor formation [42]. This was accompanied by a global upregulation of oxidative phosphorylation and glycolysis transcriptomes, causing deregulated mitochondrial respiration, oxidative stress, impaired cell proliferation, and a switch to glycolytic metabolism in vivo. These data identify TRAP-1 as a central regulator of mitochondrial bioenergetics, and this pathway could contribute to metabolic rewiring in tumors [42]. Here we illustrate how high resolution heteronuclear NMR can complement MS and can be used to determine the levels of labeling in different subunits of intact lipids on a global scale, using PC3 (prostate cancer) and UMUC3 (bladder cancer) cells with or without TRAP-1 knockout, grown in the presence of uniformly ^13^C-labeled ([U-^13^C])- glucose or [U-^13^C]-glutamine.

## 2. Results

### 2.1. NMR Analysis

High resolution tandem mass spectrometry can provide very detailed information about the particular lipid species present in a complex mixture, and thus about the labeling patterns of metabolic subunits. However, that approach is less suited for addressing questions about lipids in bulk including the major metabolic subunit labeling (e.g., glycerol versus acyl chains) and the relative abundances of classes of complex lipids (e.g., phosphatidylcholines and cholesterol). We have developed a way to quickly estimate the incorporation of ^13^C into different subunits of complex lipids in bulk by NMR. Using cellular phosphatidylcholine lipids (PCs) as an internal standard, we can quickly assess the ^13^C incorporation levels into different functional groups of unfractionated lipids. Since mammalian cells do not have the ability for *de novo* synthesis of choline, all the choline methyl groups inside the cells are at natural abundance (1.07%). The ratio of peak intensities of other species to that of PC methyl peaks in the proton spectrum represents the unlabeled species and that from the HSQC spectrum reflects the labeled species. The relations between these two ratios gives the ^13^C fractional enrichment of the specific groups. Furthermore, by comparing the intensities of the choline peaks with those of glycerol or cholesterol, the relative amounts of these classes of lipids can be estimated.

### 2.2. ^13^C Incorporation from Glucose and Glutamine by ^1^H NMR

**Figure 1:**
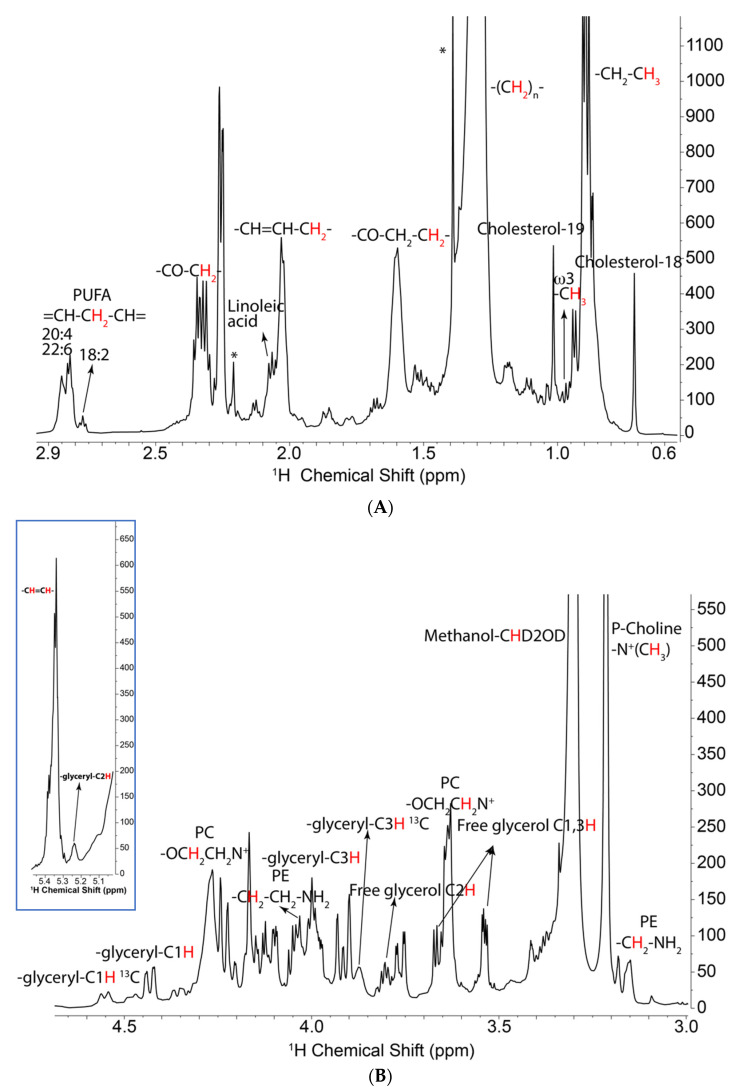
1D NMR spectra of lipids extracted from UMUC3 WT (wild type) cells. Lipids were extracted from cells grown in the presence of [U-^13^C]-glucose, dried, and redissolved in d_4_-methanol. NMR spectra were recorded at 15 °C with 1D ^1^H-NMR collected with 512 transients and an acquisition time of 2 s with 14,368 points using a spectral width of 12 ppm with a relaxation delay of 4.0 s. The data were linear predicted and zero filled to 65,536 data points and well resolved peaks are labeled as shown according to the literature [43] and our in-house standard database. ^1^H PRESAT spectrum. (**A**) Upfield region showing acyl chains and cholesterol resonances. The vertical scale is expanded to show the peaks that are small compared with that of the bulk CH_2_ resonance at 1.32 ppm. (**B**) Lower: downfield region showing glycerol and headgroup region; inset shows the double bond and glycerol C2 region. Assignments are displayed in the figure. The sharp resonances (asterisks) at 1.4 and 2.2 ppm are from BHT.

Two different cell lines (PC3 and UMUC3) were treated with either [U-^13^C]-glucose or [U-^13^C]-glutamine tracers (U = uniformly labeled). The cellular lipids were extracted and analyzed by ^1^H NMR for maximal sensitivity. However, due to the overall low fraction of ^13^C lipids species and severe overlapping with other signals, the ^13^C satellite peaks of those incorporated species are difficult to quantify. Figure 1 shows a 1D ^1^H spectrum of PC3 lipids grown in the presence of [U-^13^C]-glucose. The acyl chains are observed in the region from 0.8 to 3 ppm (Figure 1A), including the terminal methyl groups, the bulk CH_2_, and the resolved protons attached to C2, C3, and C4. In addition, cholesterol methyl resonances are well resolved at 0.7 and 1 ppm. ^13^C satellites are difficult to discern in these spectra due to their low intensity as well as overlapping with other signals. The choline and ethanolamine headgroup resonances and those of the glycerol backbone subunit of the phospholipids are between 3 and 5 ppm (Figure 1B). Only one of the glycerol head group C1 carbon satellites is easily observed in the proton spectrum (Figure 1B). The C3 carbon satellite is visible but hard to quantify, as it overlapped surrounding peaks.

**Figure 2:**
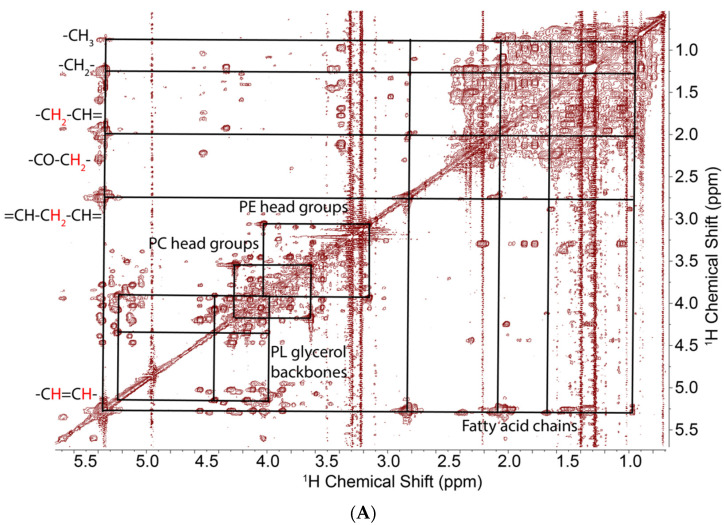

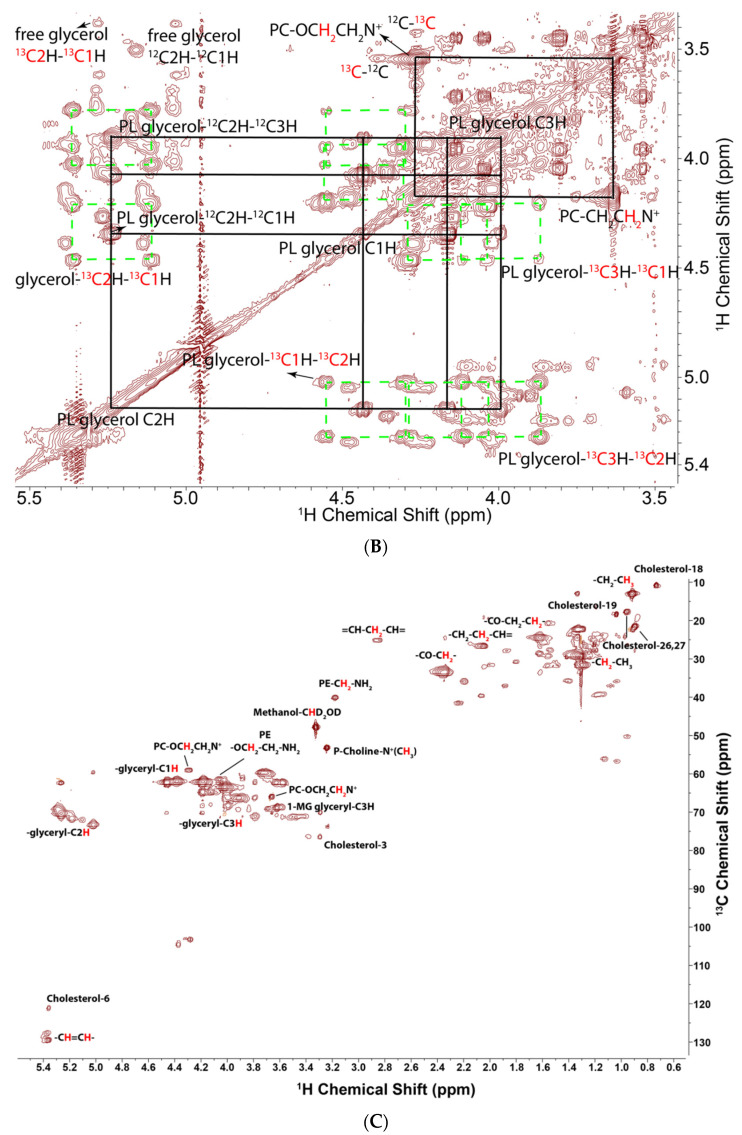
^1^H TOCSY and ^1^H{^13^C}-HSQC spectra of lipids extracted from PC3 cells grown on [U-^13^C]-glucose. TOCSY spectra were recorded at 15 °C, 14.1 T with acquisition tines of 1 s in t_2_, 0.04 s in t_1_ with a DIPSI2 (Decoupling In the Presence of Scalar Interactions) spin lock of 50 ms mixing time, and a B_1_ field strength of 6.5 kHz. The 2D HSQC spectrum was acquired with 12 ppm in the proton dimension and 200 ppm in the carbon dimension. Acquisition time is 0.25 s in F2 and 8.5 ms in F1. Adiabatic decoupling was applied and a transfer delay of 3.425 ms corresponding to 146 Hz was set for an optimal one bond CH coupling. (**A**) Full spectrum showing different subgroups of complex lipids. (**B**) Downfield region expansion showing cross peaks and ^13^C satellites in the glycerol subunits, and cross peaks of the choline headgroup. Green dashed boxes indicate the doubly labeled adjacent carbons in the glycerol backbones. (**C**) HSQC spectrum confirming the assignments from the PRESAT and TOCSY spectrum.

To determine the labeling patterns more clearly, we recorded two dimensional TOCSY and HSQC spectra, which showed clear cross peak patterns and appropriate chemical shifts for all the C1, C2, and C3 positions of the glycerol subunit in glycerolipids [44]. The TOCSY spectra (Figure 2A,B boxes) showed reproducible and substantial ^13^C labeling in the glycerol subunits (>50%, Table 1) [34,35,45] of extracts of cells grown in the presence of [U-^13^C]-glucose, which were absent in the extracts of cells grown on unlabeled media or media containing [U-^13^C]-glutamine. The HSQC spectrum (Figure 2C) confirmed the assignments of the peaks observed in the 1D and TOCSY spectra. The ^13^C satellites in the TOCSY spectrum can be volume integrated to provide the positional (isotopomer) enrichment in the glycerol subunits. The TOCSY spectra also show some resolution of the acyl chains, most notably at the sites of unsaturation, the terminal methyl group, and at the C2,C3,C4 positions of the acyl chains (Figure 2A). However, in these cells, the labeling of the acyl chains was low, such that the integration of the TOCSY and 1D proton spectra was not reliable if not impossible. We then turned to the assessment of ^13^C incorporation using 1D ^13^C{^1^H}-HSQC spectra, as described in the Methods.

### 2.3. Determination of ^13^C Incorporation from Glucose and Glutamine by ^1^H{^13^C}-HSQC

**Figure 3:**
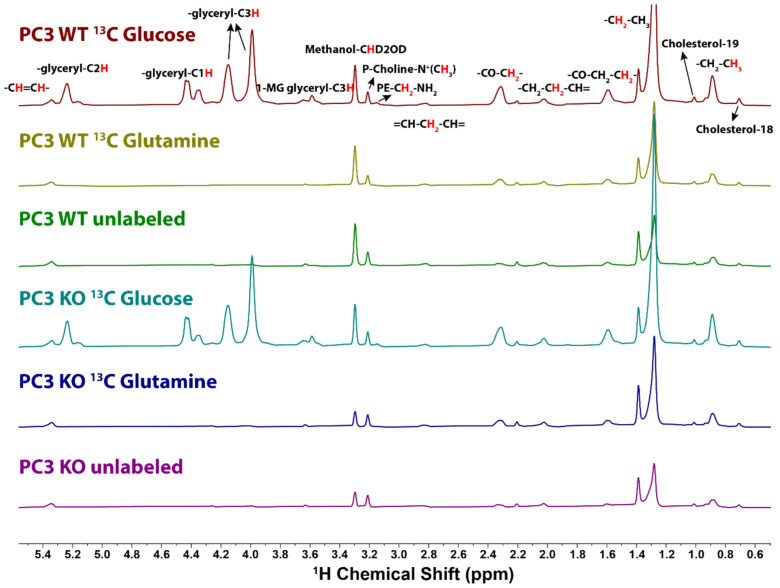
1D ^1^H{^13^C}-HSQC spectra of the lipid extracts from WT or KO PC3 cells cultured in [U-^13^C]-glucose, [U-^13^C]-glutamine, or no tracer. The spectra were recorded with ^13^C adiabatic decoupling during the acquisition time of 0.25 s for 1024 scans (34 min acquisition). The spectral width was set at 12 ppm the same as the proton spectrum and recycle delay was set to 1.75 s. A total of 1796 data points were collected and zero filled to 4096 points with a 4 Hz line-broadening exponential. [U-^13^C]-Glucose tracer samples in both cell types showed ^13^C incorporation into various functional groups, with glycerol backbones as the most heavily labeled groups. In contrast, [U-^13^C]-glutamine labeled samples only showed incorporation mainly into bulk acyl-chains as well as C2, C3 positions on the fatty acids, indicating the main contribution of *de novo* fatty acid synthesis. The 1D HSQC spectra shown are normalized to cell numbers.

The 1D HSQC spectra of the lipid extracts from PC3 and UMUC3 cells grown in the presence of either [U-^13^C]-glucose or [U-^13^C]-glutamine are compared with the same cells grown in the presence of unlabeled glucose in Figure 3 and Figure S3. As Figure 3 shows, the extracts of cells grown on [U-^13^C]-glucose show much more intense glycerol peaks than either the [U-^13^C]-Gln or unlabeled samples. Revealingly, the various acyl chain peaks are also more intense in the glucose sample indicating significant ^13^C incorporation into the fatty acyl chain. The actual ^13^C incorporation into each position was calculated, as described in the Methods section. From Table 1, it is clear that, in both cell lines grown on ^13^C-glucose, the glycerol head groups were heavily enriched (over 50%), since they derive from dihydroxyacetone-3-phosphate, which is an intermediate of glycolysis. These agreed very well with the integration from the carbon satellite peaks of these groups from proton spectra as well as 2D TOCSY spectra. We quantified the central unlabeled peaks as well as the ^13^C satellite peaks from the TOCSY spectrum (cf. Figure 2A) of one WT PC3 sample and found that the glycerol C1 group ^13^C enrichment was 60.31%, the C2 group enrichment was 51.26%, and the C3 group enrichment was 59.57%, respectively, and these are very close to the figures calculated from 1D HSQC for this particular sample (62.7%, 50.4%, and 66.7%). Furthermore, the natural abundance ^13^C level averaged over all unlabeled samples and positions was 1.08 ± 0.37% (Table 1), which agrees well with the expected value of 1.07%. This indicates that our method of estimating the fractional enrichment from the 1D HSQC is viable. For comparison, we also recorded a direct observe ^13^C spectrum of the lipid extract of PC3 cells grown in the presence of [U-^13^C]-glucose, which required 18 h acquisition for a comparable signal-to-noise ratio to the 1D HSQC (Figure S4).

We used the same method to determine the ^13^C incorporation into UMUC3 cells. At the glycerol-C1 group, we found 59.3 ± 0.9% enrichment from [U-^13^C]-glucose in the wild type cells and 52.8% ± 1.28% in the UMUC3 knockout cells, and not different from natural abundance for the [U-^13^C]-Gln samples (Table 1). The ^13^C labeling of the glycerol carbons was similar to the wt PC3 cells (62.3 ± 1.2%), but slightly lower than in the PC3 KO cells (65.2 ± 0.88%). This indicates a high fraction of the glycerolipid pool turnover using G3P newly synthesized from glucose. In comparison, labeling of the bulk CH_2_ groups in the lipid fatty acid chains was much lower, around 4–5%. Terminal methyl groups showed around 2–3% labeling, indicating that the ab initio de novo synthesis of fatty acid chains are relatively low in these cells. Meanwhile, the C2 and C3 groups which are next to the carboxylate end of the acyl chains, were more highly labeled (7–9% for C2 and 4–6% for C3) due to extension with newly synthesized [46] AcCoA from glycolysis and PDH activity.

**Figure 4:**
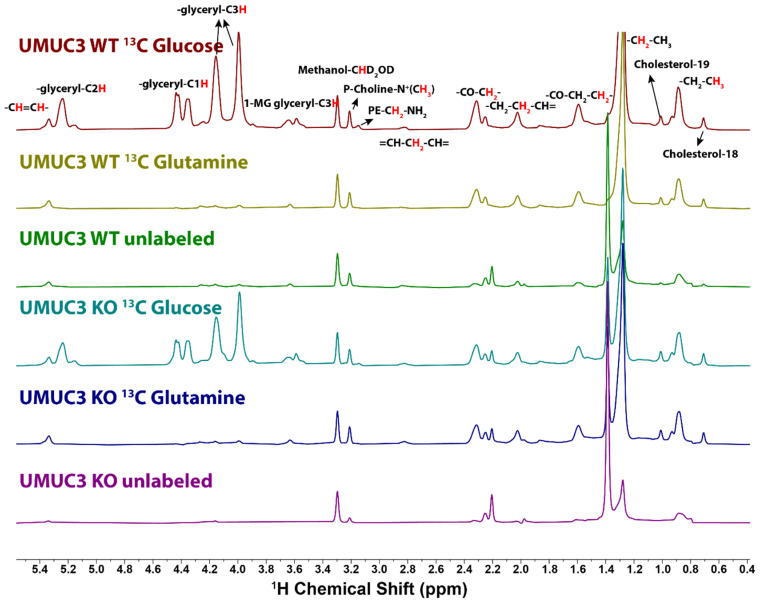
1D HSQC spectra of the lipid extracts from UMUC3 cells grown in the presence of [U-^13^C]-glucose, [U-^13^C]-glutamine and unlabeled glucose. UMUC3 cells were grown and extracted, then prepared for NMR as described in the Methods section. The HSQC acquisition parameters were as described in Figure 3.

In cells grown in the presence of [U-^13^C]-Gln and unlabeled glucose the glycerol groups showed no significant labeling (i.e., close to natural abundance) (Figure 4, Table 1), indicating little *de novo* gluconeogenesis in these cells, as previously observed for breast cancer cells [35]. However, the fatty acid chains were enriched to around 3–4% due to the cytosolic AcCoA generated from citrate that may arise from reductive glutamine metabolism and transported out of the mitochondria [47,48,49] or exchange flux [49].

In [U-^13^C]-glucose tracer experiments, the glycerol group fractional enrichments were similar in both PC3 and UMUC3 cell lines. In contrast, the bulk CH_2_ in the PC3 cells were more enriched than that in the UMUC3 cells. Both C2 and C3 of the fatty acids chain were more highly enriched in PC3 cells compared with UMUC cells, indicating a more active fatty acid extension reaction in PC3 cells. In the [U-^13^C]-glutamine tracer experiments, the labeling in the various fatty acid groups was in general lower in PC3 than in UMUC3, and in both cases was not much above natural abundance.

For the labeled samples, the cholesterol 18 and 19 methyl groups showed ^13^C enrichments above a natural abundance of 4–5% in the UMUC3 cells, and these were lower (2–3%) in the PC3 cells (Table 1), indicating a higher flux of AcCoA into cholesterol synthesis in UMUC3 cells.

### 2.4. Estimation of Lipid Distributions from NMR

In addition to measuring the ^13^C incorporation into different metabolic subunits (e.g., glycerol, acyl chains, and cholesterol), the NMR spectra also provide information about the distribution of classes of lipids. For example, the relative amount of PCs in the total glycerolipid pool could also be estimated from the ratio of the glycerol group to the choline methyl group. Thus, the glycerol resonances represent all glycerol lipids, whereas the choline NMe^3+^ resonance at 3.21 ppm represents the total PC levels. From the unlabeled samples and the samples grown in the presence of [U-^13^C]-Gln, we estimate that the PC/total GPL was 55.2% ± 5.6% for WT PC3 and 39.3% ± 6.1% for UMUC3.

Using the resolved methyl resonances of cholesterol at 0.71 and 1.02 ppm in both ^1^H and HSQC spectra (Figure 2), we determined the ratio of lipid choline to cholesterol in 1D ^1^H spectra. For the unlabeled samples, we estimated the choline/cholesterol ratio as 1.85 ± 0.24 for UMUC3 WT and 1.65 ± 0.05 for PC3 WT.

The TOCSY spectra (Figure 2B) also show the presence of other headgroups, notably ethanolamine, which is therefore relatively abundant. Comparing the intensity of the methylene peaks from PE (Phosphatidylethanolamine) head groups and PC headgroup peaks showed a PC/PE ratio of 1.2 in the PC3 WT cells. However, we observed no significant intensity from PS in these samples, though such lipids were observed in mass spectra of the samples, indicating that this class of lipids is at low overall abundance.

## 3. Discussion

NMR has been applied for lipidomics analysis, and with some isolated peaks in the proton spectrum, the quantification of certain types of lipids can be made from a series of equation manipulations in standard mixtures or lipid hydrolysis products [50,51]. However, for crude lipid extracts, due to the complexity of the lipid structure and species, few NMR methods have been applied this type of study, let alone with isotope tracers. Here we report a different perspective to apply NMR for lipidomics studies. Instead of quantifying individual lipid species, we investigated the overall substructure of the lipid functional groups and traced the incorporation of ^13^C from [U-^13^C]-glucose or [U-^13^C]-glutamine.

PC is the most abundant lipid class in mammalian cells [52] and is highest in cells that do not contain significant lipid droplet stores [53]. It may also be impacted in certain cancers [54]. We have used 1D ^1^H NMR and 2D TOCSY and HSQC to characterize the metabolic labeling of abundant lipid classes in two cancer cell lines. From the TOCSY spectra, we were able to determine the ^13^C incorporation into the glycerol subunits with a CoV of <5% (at a labeling level of >50%). For lower level incorporation, where the HSQC spectra were used, the estimated CoV were <10% for the acyl chains and 5–7% for the cholesterol methyl groups. For the unlabeled samples, the ^13^C level at each site should be the natural abundance level, i.e., 1.07%. Table 1 shows that, whereas most peaks are close to the expected value, with an overall CoV of 5–10%, there are a couple of outliers, which likely reflect integrations at low total abundance and/or signal overlap. These values place a lower limit on estimating enrichment of 2–4%.

In both cell lines, the glycerol subunit, which is predominantly PC, was heavily labeled from glucose but not from glutamine. In contrast, the acyl chains and cholesterol were much less extensively labeled, similar to HEK293 cells [34] or MDAMB231 cells [35] but unlike in some other cell lines [20,35,46,55,56]. The high enrichment in the glycerol subunit indicates substantial *de novo* lipid synthesis using glucose via DHAP/G3P, but relatively little glucose or Gln contributed to the AcCoA pool, as the acyl chains and cholesterol were enriched about one order of magnitude less. As the cells proliferate and make new lipids, either these cells recycle fatty acids (such as from internal lipid stores [34]), take up fatty acids from the medium [57,58], or use carbon sources other than glucose or Gln to generate AcCoA.

In general, PC comprises 40–50% of total phospholipids, while PE comprises around 15–25% in mammalian cells and subcellular organelles [52]. The PC fraction in the PC3 wild type cells was 55.2% ± 5.6% and 53.8 ± 7.3% for KO cell lines, whereas the enrichment was 39.2% ± 6.1% for UMUC3 WT cells and 49.4% ± 15.1% for KO cells.

The cholesterol/GPL ratio was also estimated from the ratios of labeled and unlabeled free cholesterol to phosphatidylcholine. According to our calculations, the total ratios of phosphatidylcholine to free cholesterol was 1.65 ± 0.05 for WT PC3 cells and 1.47 ± 0.08 for KO PC3 cells, whereas these ratios were 1.85 ± 0.24 and 1.62 ± 0.15 for WT and KO UMUC-3 cells, respectively. This compares with the PC/free cholesterol ratio in mouse livers of 2.5–2.7 on a regular diet and 1.0–1.1 on a high cholesterol diet [59].

PC can be generated from the CDP-choline pathway or PE N-methyltransferase (PEMT) pathway, where PE is converted into PC by three methylation reactions. Mouse hepatic PC/PE ratio between 1.5 and 1.8 maintains membrane integrity, while a ratio of <1 leads to membrane destruction [60]. Quantification of cross peaks of head groups of PC and PE from the TOCSY spectrum showed the PC/PE ratio is around 1.2 for PC3 WT cells. We did not observe significant amounts of PS in either 1D or 2D NMR, indicating the low abundance of PS in these two types of cancer cells. We also observed insignificant labeling of the PE head group, suggesting that glucose was not a major source of serine carbon in these cells.

## 4. Materials and Methods

Wild type PC-3 (prostate cancer) and UMUC3 (bladder cancer) cells were purchased from ATCC (American Type Culture Collection, Manassas, VA, USA), and the TRAP-1 KO version was generated in-house in the laboratory of Dr. L. Neckers. Cells were seeded in 10 cm dishes at 3 million cells per dish, incubated in either a DMEM (Dulbecco’s Modified Eagle’s Medium) medium containing 25 mM [U-^13^C]-glucose, 2 mM unlabeled Gln (in triplicate), 25 mM unlabeled Glc + 2 mM [U-^13^C,^15^N]-Gln and 10% dialyzed FBS (in triplicate), or unlabeled media (single plate), at 37 °C in a 5% CO_2_ atmosphere, RH > 90% for 24 h (approximately one cell doubling). After incubation, the medium was aspirated, and the cells (6 million per plate) were washed twice with ice cold PBS and then quenched on the plate as previously described [3]. Metabolites were extracted using a modified Folch method [61], and the organic layer was dried in a vacufuge at room temperature and resuspended in 2:1 (*v*/*v*) chloroform/methanol + 1 mM Butylated HydoxyToluene (BHT) for storage at −80 °C prior to NMR analysis [3].

NMR analysis: Lipid samples were dried again prior to the NMR experiment and reconstituted in 100% methanol-d4 (CIL, Tewkesbury MA) and loaded into 3 mm matched Shigemi NMR tubes. 1-D proton (PRESAT), TOCSY (zTOCSY) and ^1^H{^13^C} HSQC spectra (gHSQCAD) were recorded with adiabatic decoupling at 15 °C on an Agilent (Santa Clara, CA, USA) DD2 14.1 T NMR spectrometer equipped with a 3 mm inverse triple resonance cold probe. Spectra were referenced to the methanol resonance at 3.3 ppm and the phosphatidyl choline NMe^3+^ resonance at 3.21 ppm. 1D ^1^H spectra were recorded with an acquisition time of 2 s with continuous weak irradiation of the HOD peak during a relaxation delay of 4 s and 512 transients (52 min). TOCSY spectra were recorded with acquisition times of 1 s in t_2_ and 70 ms in t_1_ with an isotropic mixing time of 50 ms at a B_1_ field strength of 6.6 kHz using DIPSI-2, with 8 transients per t_1_ increment and 512 complex pairs (4.6 h acquisition). Assignments and isotopomer distributions in ^1^H NMR were determined as previously described [20,35,36,62]. HSQC spectra were recorded with adiabatic ^13^C decoupling during a 0.25 s acquisition time, with a relaxation delay of 1.75 s and 1024 transients (34 min acquisition). The 1D ^13^C gated decoupler experiment was recorded in 18 h using the X-coil of the 3 mm probe as the HSQC, with an acquisition time of 1 s, a relaxation delay of 1 s, and 32,000 transients.

Isotopomer distributions were also determined from 1D ^1^H{^13^C}-HSQC by comparing the relative integrals (areas) of peaks with that of the choline N(CH_3_)_3_ resonance at 3.21 ppm, which is exclusively natural abundance, with the same ratio in an unlabeled sample. A higher ratio indicates ^13^C incorporation, and the enrichment is directly proportional to the ratios in the two sample types.

Positional enrichments, F, were calculated from TOCSY and 1D spectra as previously described [63].

(1) $$F=\frac{A\left({}^{13}C\right)}{A\left({}^{13}C\right)+A\left({}^{12}C\right)}$$

where A(^13^C) is the area of the ^13^C satellites, and A(^12^C) is the area of the unlabeled peak.

For low level enrichments, the positional ^13^C fractions for each lipid group by HSQC, F was determined from the 1D ^1^H and 1D HSQC spectra using

(2) F=0.0107∗A(13Cx)HSQCA(13Cc)HSQC0.0107∗A(13Cx)HSQCA(13Cc)HSQC+0.9893∗A(12Cx)1HA(12Cc)1H

where A(^13^C_X_) _HSQC_ and A(^13^Cc) _HSQC_ are the areas of peak X and the choline methyl resonances in HSQC spectra, and A(^12^C_X_) _1H_ and A(^12^Cc) _1H_ are the areas of peak X and the choline methyl resonances in the ^1^H PRESAT spectrum.

In the PC/total lipids or PC/cholesterol ratio calculation, we used the glycerol-C3H and cholesterol-18 methyl group peak as representative signals. Since their enrichment levels are different (PC is only natural abundance), both ^13^C and ^12^C species contribute to the total ratio but at different weights. Here, x represents either cholesterol or glycerol-C3H, while c represents choline.

The ratio of PC to total lipids or to cholesterol, R, can be described as follows:

(3) R=10.9893∗A(12Cx)1HA(12Cc)1H+0.0107∗A(13Cx)HSQCA(13Cc)HSQC

Area notations are the same as described above.

## 5. Conclusions

The methods described here provide both high resolution mapping of complex lipids without chromatographic separation and a direct way of generating an overview of ^13^C incorporation into bulk lipid molecules, which can be utilized as a standalone approach or to complement targeted mass spectrometry-based lipidomics workflows [45]. Further, with a detection limit as low as 2% with a COV of <10% by HSQC, it provides very valuable metabolic information that other techniques cannot easily generate. HSQC is sufficient to provide both high and low labeling isotopomer quantification. The TOCSY spectra are useful for validating peak assignments, for improved resolution and satellite quantification when ^13^C incorporation is relatively high (cf. the glycerol subunits in the present study), and for cross-validating HSQC analyses under those conditions. The sensitivity advantage of proton detection over ^2^H or ^13^C enables estimation of low (<10%) ^13^C enrichment or with much smaller quantities of material.

Although we demonstrated the method here using 2D cell culture, we and others have successfully applied the SIRM approach to a variety of different systems, including 3D spheroids, mouse models, and human subjects. Introducing a label via injection or diet is straightforward [35,62,64,65,66], and the extraction procedures for tissues are also well established. From then on, the analysis is the same as for cells. We note also that, with the amount of material used in this study, the necessary ^1^H and 1D HSQC spectra can be recorded in about 1.3 h per sample using a cryoprobe, implying an approximate throughput of up to 20 samples per day under automation, without destruction of the samples. In contrast, direct observation of ^13^C using a cryoprobe would have a throughput of 1–2 samples per day.

## Figures and Tables

**Table 1:** Summary of fractional ^13^C level in lipid subgroups from both cell lines (WT: wild type as well as KO: knock out) determined by NMR. Lipid subgroup ^13^C levels were calculated as described in the text (Equations (1) and (2)) and are presented as % of the total carbon at different position in the lipids (mean ± standard deviation for triplicate labeled samples, only one unlabeled sample in each group).

Groups	UMUC 3						PC3					
	Glc		Gln		Unlabeled		Glc		Gln		Unlabeled	
	WT	KO	WT	KO	WT	KO	WT	KO	WT	KO	WT	KO
Acyl CH_2_	2.7 ± 0.43	3.0 ± 0.60	2.8 ± 0.37	3.9 ± 0.39	1.73	1.28	3.8 ± 0.24	5.4 ± 0.29	1.8 ± 0.20	1.45 ± 0.07	1.0	0.86
Methyl– CH_2_–CH_3_	3.1 ± 0.15	2.45 ± 0.16	2.7 ± 0.33	2.3 ± 0.29	1.81	2.18	2.6 ± 0.07	3.1 ± 0.07	1.5 ± 0.01	1.5 ± 0.11	0.94	1.08
Acyl CH_2_–CO	5.2 ±0.26	4.9 ± 0.42	3.9 ± 0.21	3.7 ± 0.18	0.81	1.34	7.4 ±0.28	8.5 ± 0.52	3.2 ± 0.03	3.1 ±0.07	0.94	1.08
Acyl CO–CH_2_–CH_2_	3.1 ± 0.19	2.5 ± 0.05	2.3 ± 0.09	2.5 ± 0.33	1.24	0.39	3.9 ± 0.14	5.5 ± 0.22	2.0 ± 0.06	2.1 ± 0.20	0.95	0.91
Glyceryl–C1H	59.3 ± 0.90	52.8 ± 1.28	nd ^a^	nd	nd	nd	62.3 ± 1.17	65.2 ± 0.88	nd	nd	0.80	nd
Glyceryl–C2H	41. 9 ± 0.55	33.4 ± 0.41	nd	nd	nd	nd	48.9 ± 1.70	53.0 ± 2.93	nd	nd	1.45	nd
Glyceryl–C3H	61.5 ± 6.81	50.4 ± 3.22	1.84	2.17	1.84	1.1	67.1 ± 0.41	72.8 ± 1.63	2 ± 0.08	2.1 ± 0.41	1.76	1.87
Acyl =CH–	1.2 ± 0.11	1.3 ± 0.03	1.1 ± 0.07	0.91 ± 0.07	0.88	1.04	0.90 ± 0.08	0.99 ± 0.06	0.88 ± 0.04	0.97 ± 0.03	0.83	0.79
Acyl =CH–CH_2_–CH=	0.97 ± 0.18	1.11 ± 0.14	1.03 ± 0.22	1.08 ± 0.14	0.97	0.89	0.77 ± 0.05	0.94 ± 0.11	0.78 ± 0.04	0.85 ± 0.05	0.89	0.70
Acyl CH_2_–CH_2_–CH=	2.2 ± 0.07	1.5 ± 0.03	1.8 ± 0.15	1.5 ± 0.06	0.74	0.45	1.8 ± 0.54	2.0 ± 0.06	0.96 ± 0.04	1.10 ± 0.06	0.93	0.90
Chol–18	5.5 ± 0.33	4.6 ± 0.04	4.4 ± 0.69	4.0 ± 0.36	1.46	nd	3.6 ± 0.08	3.2 ± 0.23	2.1 ± 0.10	1.9 ± 0.09	1.54	1.38
Chol–19	3.8 ± 0.24	3.6 ± 0.11	2.6 ± 0.14	3.3 ± 0.15	0.68	nd	2.7 ± 0.04	2.1 ± 0.07	1.7 ± 0.08	1.4 ± 0.16	0.85	1.36

^a^ not determined.

## Data Availability

Original data are available from the authors on request.

## Supplementary Materials

The following are available online at https://www.mdpi.com/article/10.3390/metabo11040202/s1, Scheme S1: Complex lipid subunits from glucose and glutamine, Figure S1: Common major lipid classes, Figure S2: Comparison of 1D PRESAT spectra of WT or TRAP-1 KO cell extracts; Figure S3: Comparison of 1D ^13^C HSQC spectra of WT UMUC3 or PC3 cell extracts; Figure S4: Carbon 1D spectrum of the lipid extract from PC3 KO cell line.
