# Supplementary material for: NMR Methods for Determining Lipid Turnover via Stable Isotope Resolved Metabolomics
Source: Metabolites. 2021 Mar 29;11(4):202. doi: 10.3390/metabo11040202 (PMC8065598; doi:10.3390/metabo11040202)

Supplementary Figures

Scheme S1. Complex lipid subunits from glucose and glutamine.
Mitochondrial AcCoA derives either from glycolysis-derived pyruvate or from Gln by reductive carboxylation, or via the Krebs cycle to malate and malic enzyme. Citrate is transported to the cytoplasm and cleaved to OAA and AcCoA by ACLY and converted to palmitate by the action of FASN. G3P derives from glycolysis via dihydroxyacetonephosphate (DHAP) and provide the glycerol subunit of glycerolipids. Red dots represent ^{13}C atoms.
ACLY ATP dependent citrate lyase; ChoP phosphocholine; FASN fatty acid synthase; GPDH glycerol phosphate dehydrogenase; ME malic enzyme; PDH pyruvate dehydrogenase; RC reductive carboxylation.

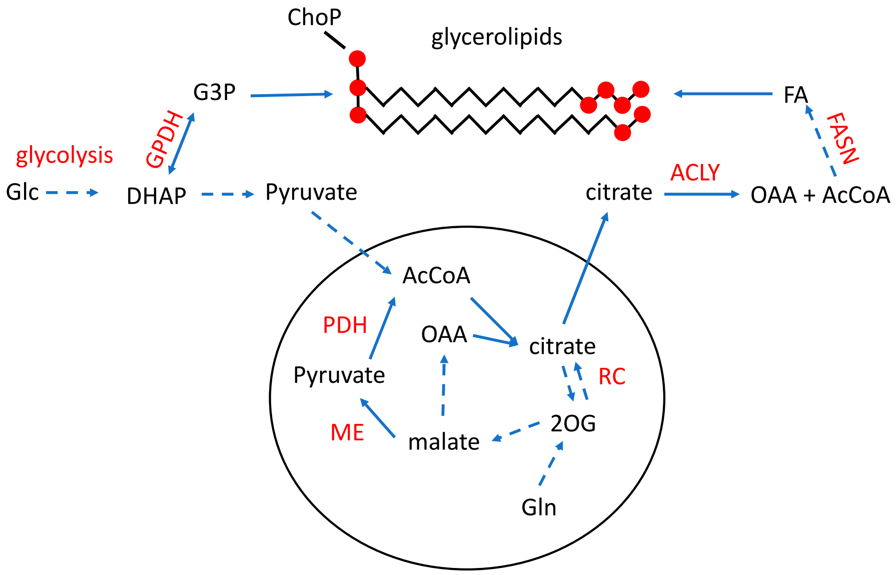

Figure S1. Common major lipid classes

The general structure of phospholipids with different headgroups are shown, along with cholesterol, the precursor of sterols and bile acids, and is frequently esterified.

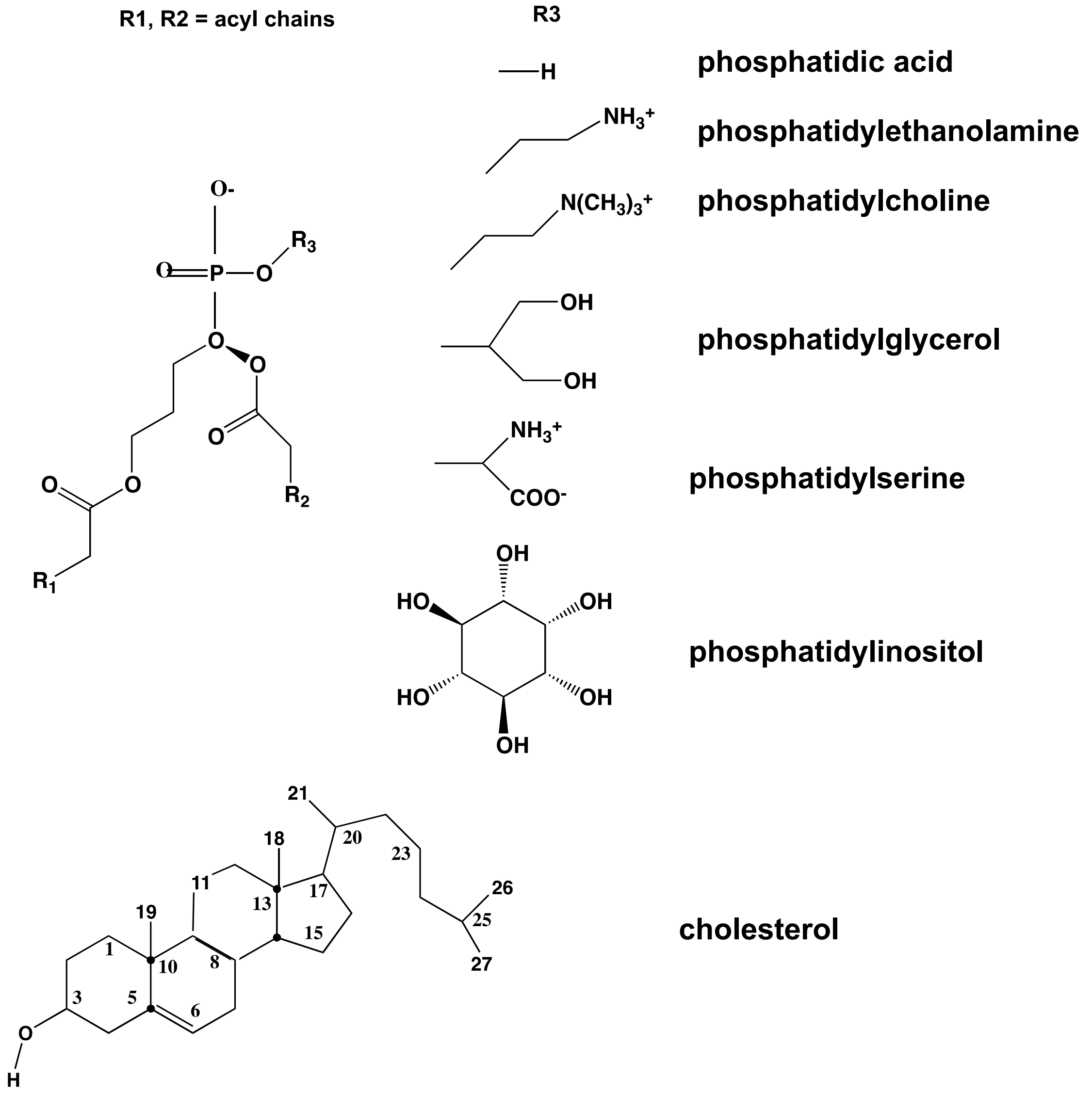

Figure S2 Comparison of 1D PRESAT spectra of WT or TRAP-1 KO cell extracts.
PC3 (A) or UMUC3 cell (B) extracts with ^{13}C glucose or ^{13}C glutamine tracers or unlabeled reference. Carbon satellites are visible in the ^{13}C glucose labeled cell extract spectra. All spectra were acquired with 3 mm MeOD matched shigemi tubes at 288 K at 14.1 T. The acquisition time was 2 s with a recycle delay of 4 s, during which a weak pulse was radiating to suppress water signals. Spectra were processed using Mnova software.

1D proton spectra of PC3 cells cultured in either ^{13}C glucose, ^{13}C glutamine or natural abundance nutrients. It is hard to find the ^{13}C incorporation from the proton spectra as the ^{13}C satellites are small and overlapping with other signals. The only visible ^{13}C satellites are one of the glycerol-C1H and -C3H satellites. Also the reduced intensities in the ^{12}C glycerol groups in the ^{13}C glucose tracer spectrum can be observed. * indicates signals from the added antioxidant BHT.

A.

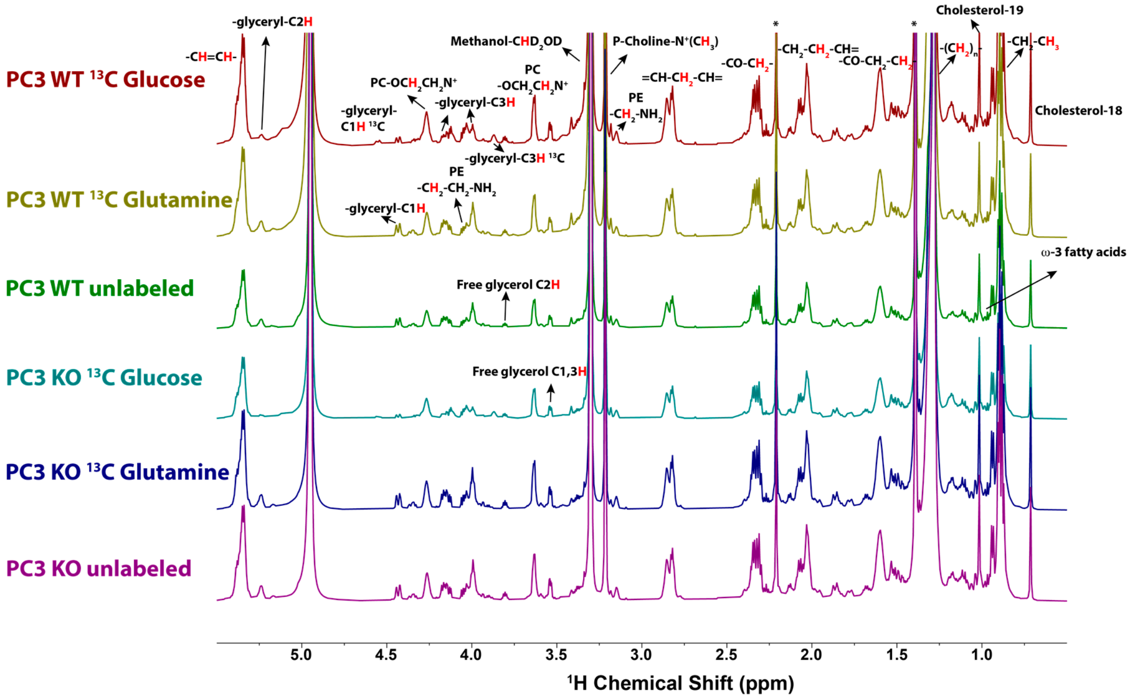

B.

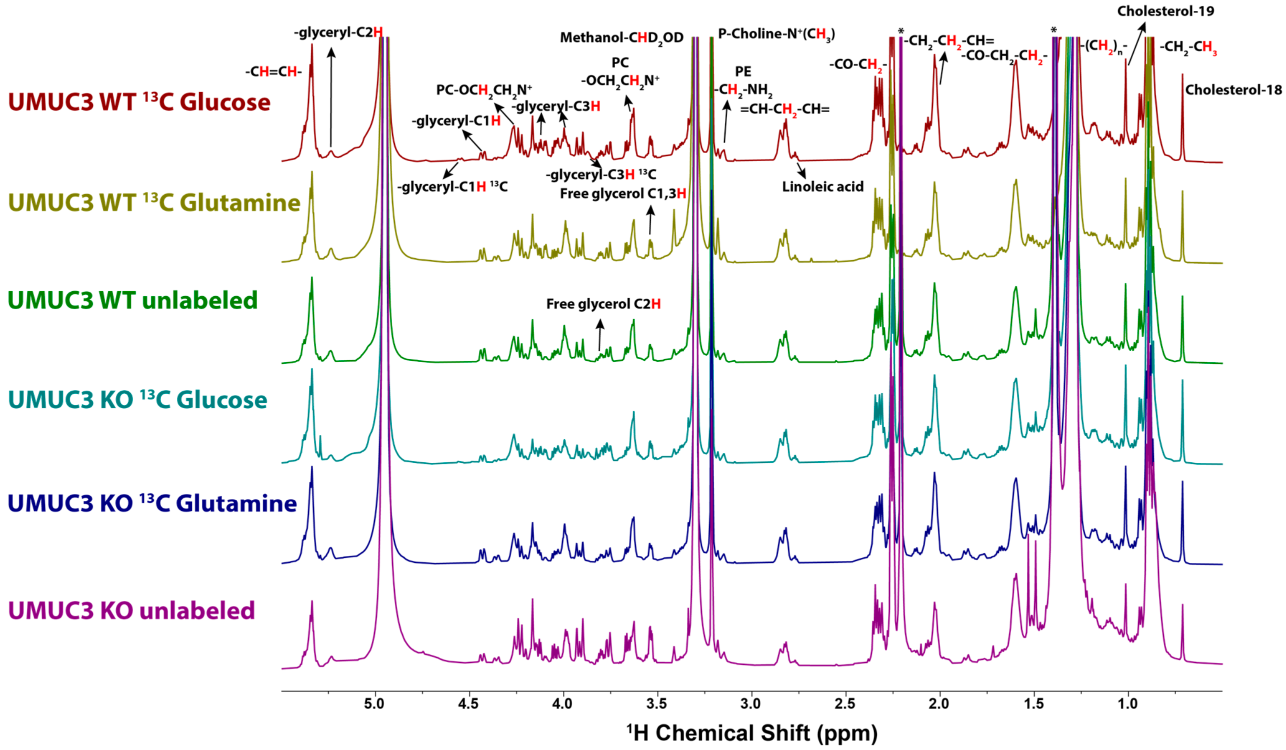
UMUC3 WT 13C Glucose
UMUC3 WT 13C Glutamine
UMUC3 WT unlabeled
UMUC3 KO 13C Glucose
UMUC3 KO 13C Glutamine
UMUC3 KO unlabeled
-glyceryl-C2H
-CH=CH-
PC-OCH2CH2N+
-glyceryl-C1H
-glyceryl-C3H
-glyceryl-C1H 13C
-glyceryl-C3H 13C
Free glycerol C1,3H
Free glycerol C2H
Methanol-CHD2OD
PC
-OCH2CH2N+
P-Choline-N+(CH3)
PE
-CH2-NH2
=CH-CH2-CH=
Linoleic acid
-CO-CH2-
-CH2-CH2-CH=
-CO-CH2-CH2-
-(CH2)n-
Cholesterol-19
-CH2-CH3
Cholesterol-18
5.0
4.5
4.0
3.5
3.0
2.5
2.0
1.5
1.0
1H Chemical Shift (ppm)

Figure S3. Comparison of 1D ^{13}C HSQC spectra of WT cell extracts
A PC3 (or B UMUC3 . PC3 and UMUC3 cells grown in the presence of either ^{13}C glucose or ^{13}C glutamine tracers and unlabled media and prepared for NMR analysis as described in the Methods. ^{13}C Glucose tracer samples in both cell types showed ^{13}C incorporation into various functional groups, with glycerol backbones as most heavily labeled groups. In contrast, ^{13}C glutamine labeled samples only showed incorporation mainly into bulk acyl-chain groups and C2 and C3 positions on the fatty acid chains, indicating the contribution of de novo fatty acid synthesis. * indicates signals from the added antioxidant BHT.

A

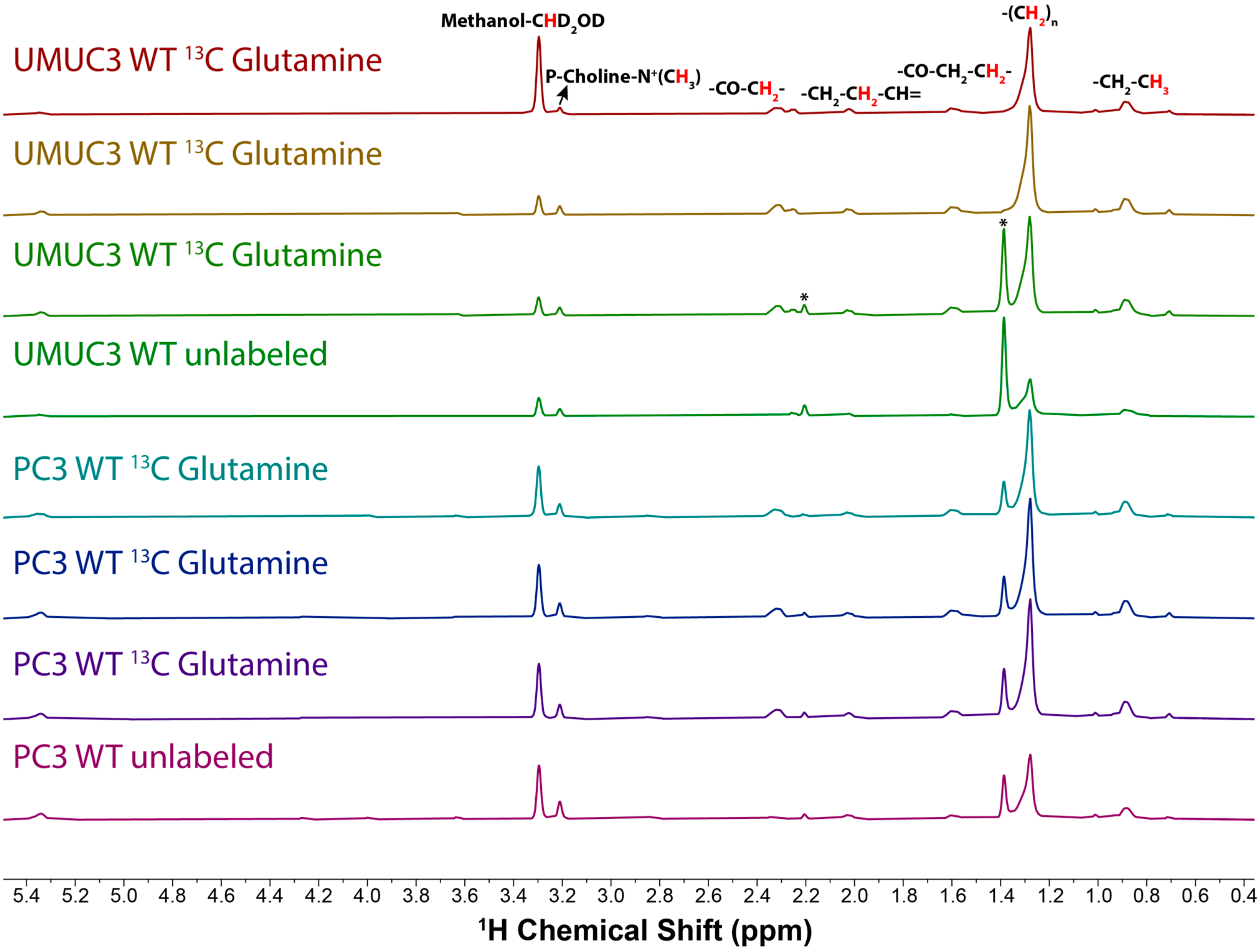

B

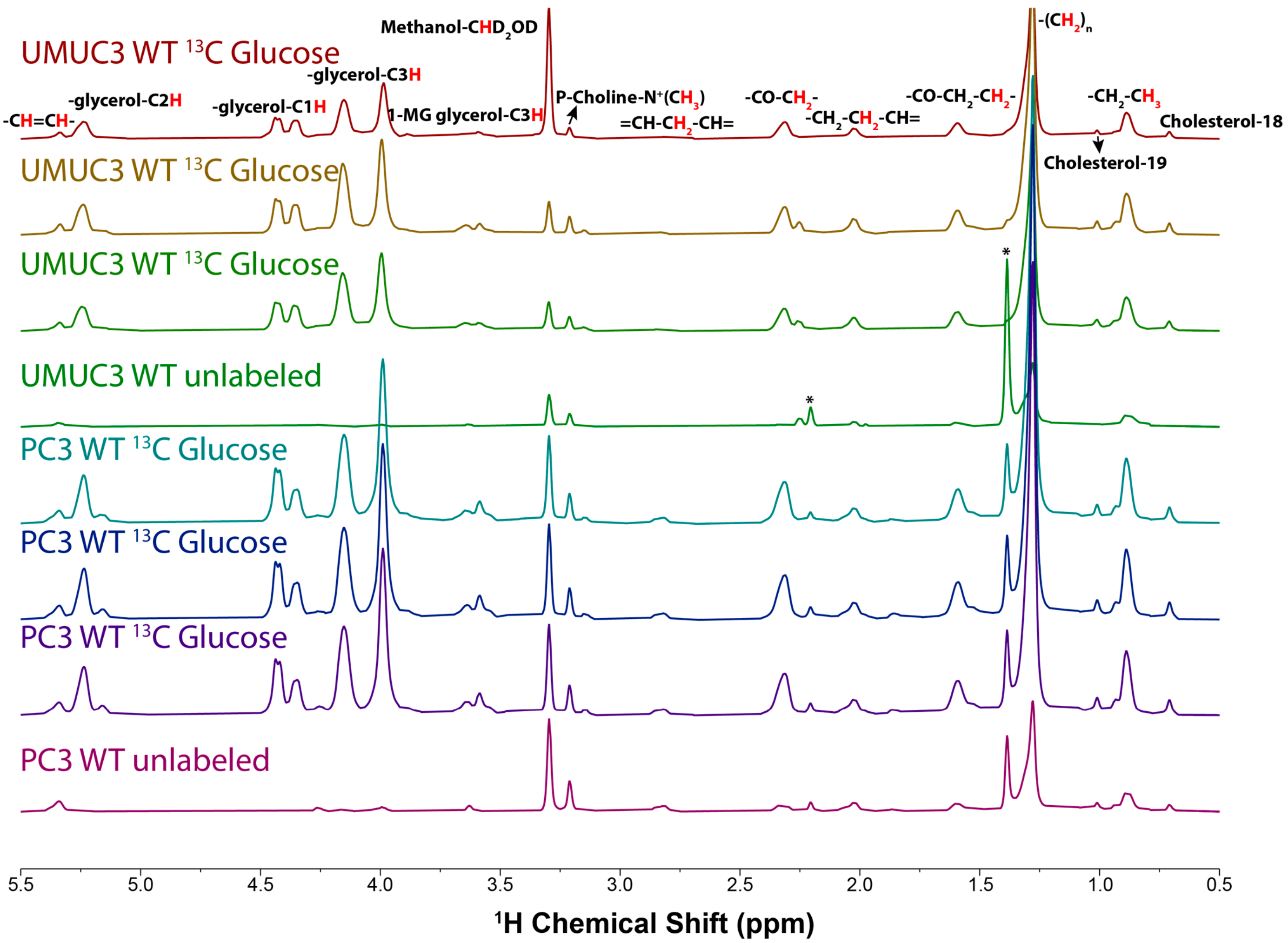

UMUC3 WT 13C Glucose
UMUC3 WT 13C Glucose
UMUC3 WT 13C Glucose
UMUC3 WT unlabeled
PC3 WT 13C Glucose
PC3 WT 13C Glucose
PC3 WT 13C Glucose
PC3 WT unlabeled
Methanol-CHD2OD
-glycerol-C3H
-glycerol-C2H
-glycerol-C1H
-CH=CH-
1-MG glycerol-C3H
P-Choline-N+(CH3)
=CH-CH2-CH=
-CO-CH2-
-CH2-CH2-CH=
-CO-CH2-CH2-
-(CH2)n
-CH2-CH3
Cholesterol-18
Cholesterol-19
*
*
5.5
5.0
4.5
4.0
3.5
3.0
2.5
2.0
1.5
1.0
0.5
1H Chemical Shift (ppm)

Figure S4. Carbon 1D spectrum of the lipid extract from PC3 KO cell lines.
The ^{13}C NMR spectrum was acquired with 45 degree flip angle with proton decoupling during the 1 s acquisition and a recycle delay without decoupling of 1 s. A total of 32,000 transients were acquired corresponding in ~18 hours acquisition. The doublet structures of the lipid terminal methyl goup indicates that majority of the detected ^{13}C methyl groups are also labeled at the neighboring CH_2 group due to the glucose-derived $^{13}C_2$-acetyl-CoA adding two carbon groups simultaneously. Most of the glycerol peaks from different lipid species are well separated. The C2 carbon peaks from glycerol all showed a triplet structure and the C1, C3 showed doublet structure due to the $^{13}C^{13}C$ coupling in the uniformly labeled glycerol backbone. The inset displays the double bond and carbonyl region at 5x magnification.

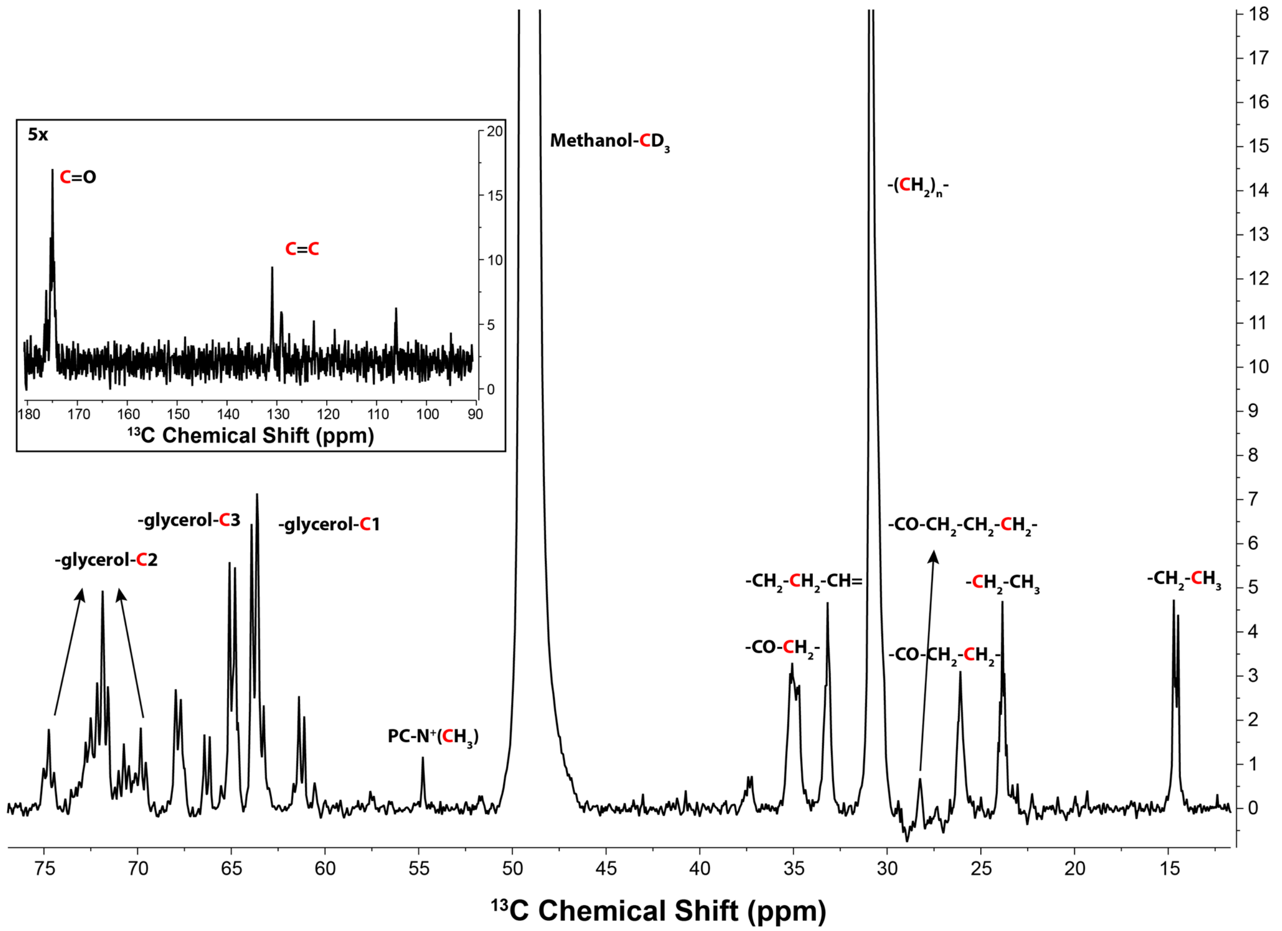

Supplement: Supplementary file 1 [file metabolites-11-00202-s001.pdf]
